# Management of severe acute malnutrition in children under 5 years through the lens of health care workers in two rural South African hospitals

**DOI:** 10.4102/phcfm.v10i1.1547

**Published:** 2018-01-30

**Authors:** Moise Muzigaba, Brian van Wyk, Thandi Puoane

**Affiliations:** 1 Faculty of Community and Health Sciences, School of Public Health, University of the Western Cape, South Africa

## Abstract

**Background:**

Despite the widespread implementation of the World Health Organization (WHO) guidelines for the management of severe malnutrition in South Africa, poor treatment outcomes for children under 5 years are still observed in some hospitals, particularly in rural areas.

**Objective:**

To explore health care workers’ perceptions about upstream and proximal factors contributing to poor treatment outcomes for severe acute malnutrition in two district hospitals in South Africa.

**Methods:**

An explorative descriptive qualitative study was conducted. Four focus group discussions were held with 33 hospital staff (senior clinical and management staff, and junior clinical staff) using interview guide questions developed based on the findings from an epidemiological study that was conducted in the same hospitals. Qualitative data were analysed using the framework analysis.

**Findings:**

Most respondents believed that critical illness, which was related to early and high case fatality rates on admission, was linked to a web of factors including preference for traditional medicine over conventional care, gross negligence of the child at household level, misdiagnosis of severe malnutrition at the first point of care, lack of specialised skills to deal with complex presentations, shortage of patient beds in the hospital and policies to discharge patients before optimal recovery. The majority believed that the WHO guidelines were effective and relatively simple to implement, but that they do not make much difference among severe acute malnutrition cases that are admitted in a critical condition. Poor management of cases was linked to the lack of continuity in training of rotating clinicians, sporadic shortages of therapeutic resources, inadequate staffing levels after normal working hours and some organisational and system-wide challenges beyond the immediate control of clinicians.

**Conclusion:**

Findings from this study suggest that effective management of paediatric severe acute malnutrition in the study setting is affected by a multiplicity of factors that manifest at different levels of the health system and the community. A verificatory study is encouraged to collaborate these findings.

## Background

The prevalence of undernutrition in children under 5 years of age remains particularly high in Africa and South East Asia.^1^ Close to 35% of the 7.6 million deaths that occur each year among children who are under 5 years of age are because of nutrition-related factors,^2^ and about 5% of such deaths are specifically attributable to severe wasting.^3^ Severe acute malnutrition (SAM) – which results from a relatively short duration of nutritional deficits^4^ – can be complicated by concurrent infective illnesses, particularly acute respiratory infection, diarrhoea and gram-negative septicaemia,^5^ as well as chronic infections such as HIV.^6^ Compelling evidence exists on the link between HIV infection and SAM, and some studies have reported high HIV prevalence among children treated for SAM.^7,8,9^ A recent study conducted in two rural South African hospitals also recorded an HIV prevalence of 43.2% in a sample of 454 children under 5 years of age who were admitted and treated for SAM.^10^

Children with SAM are much more likely to die, with or without complications, than their well-nourished counterparts.^11^ Furthermore, studies conducted in Africa have shown that children with SAM who are co-infected with HIV are at increased mortality risk compared to their HIV uninfected counterparts.^12,13,14,15,16^ In a bid to improve mortality outcomes for children with SAM, the World Health Organization (WHO) developed ‘10 steps’ guidelines for the management of SAM, which are currently widely promoted as the standard by which severely malnourished children should be treated.^17^ With strict adherence to these guidelines, mortality can be reduced to less than 5%.^18^ Some studies have shown that the implementation of these guidelines can be feasible and sustainable even in small district hospitals with limited resources.^19,20,21^ However, two recent studies conducted in the same setting have shown that improved case fatality rates (CFRs) are not always sustained over time.^22^ Furthermore, the risk of death and poor nutritional recovery remained substantially high among SAM cases with HIV infection and other baseline comorbidities despite consistent implementation of the WHO 10-step guidelines.^10^ This study was prompted by findings from the abovementioned two studies and sought to establish, from the perspectives of the health care workers (HCWs), what drives poor treatment outcomes in these two rural district hospitals located in the Eastern Cape Province in South Africa. This article describes HCWs’ attitudinal and perceptual issues and experiences that may influence clinical practice and care for children admitted for SAM with or without other comorbidities in these settings.

## Methods

### Description of the study settings

This study was carried out in two rural district hospitals located in the Eastern Cape Province in South Africa. The province is situated in the former Transkei, an Apartheid-era homeland and one of the most under-resourced regions in South Africa.^23^ A study conducted in the same hospitals^10^ showed that the prevalence of HIV infection among children aged between 6 and 60 months who were admitted between 2009 and 2013 for SAM was 43.2% with a high CFR of 24.4% in both hospitals. The study also revealed that during this period, admissions related to SAM in both hospitals constituted – on average – 50% of the total ward admissions in the paediatric ward.

### Study design

A descriptive qualitative research was conducted to describe HCWs’ perceptions about the factors that contributed to poor treatment outcomes for SAM cases in the hospitals where they worked.

### Participants

The study involved a purposive sample of HCWs who were working at the two district hospitals at the time of the study. In each hospital, two groups of HCWs were identified to take part in focus group discussions (FGDs). One group consisted of a mixture of the senior clinical personnel and senior staff from the hospital’s clinical management cluster. The other group consisted mostly of relatively junior clinical staff in the nursing category. Table 1 outlines the number and designations of the participants who took part in the FGDs at each hospital.

**TABLE 1:** List of participants from each hospital who took part in the focus group discussions.

Variables	Number of participants
**Hospital A**	
Senior clinical and management team	
Medical Officer (MO)	1
Dietician	1
Pharmacist	1
Social worker	1
Nursing service manager	1
Paediatric nurse	1
Assistant manager – nursing	1
Area manager – maternity	1
Infection control coordinator	1
Operations manager on behalf of the chief executive officer (CEO)	1
Junior clinical staff	
Staff nurse	4
Junior nurse	1
Professional nurse	2
**Hospital B**	
Senior clinical and management team	
Nursing service manager	1
Medical Officer (MO)	2
Social worker	1
Dietician	1
Pharmacist	2
Deputy director – clinical services	1
Quality assurance officer	1
Operations manager	1
Hospital administrator	1
Junior clinical staff	
Ward nurse	3
Student nurse	1
Professional nurse	1
**Total**		**33**

### Data collection

Before data collection, a personalised invitation letter to participate in the study was sent to each participating hospital, 1 month ahead of data collection. The letter was addressed to the hospital manager or chief executive officer (CEO) and specified the purpose of the study, the profile of the participants required and the scheduled dates and times for each FGD session.

On the day of data collection, all study participants received information sheets explaining the purpose of the research and what the FGDs would entail. Participants were also requested to voluntarily hand-sign a consent form before taking part in the study. The FGDs were structured and conducted as per the guidelines by Krueger.^24^ All the discussions were held in English, a language that all participants spoke and understood well. The discussions started with an overview of the performance of the concerned hospital regarding clinical outcomes as per the results of two epidemiological studies published elsewhere.^10,22^ The overview was then followed by a series of open-ended questions designed to tap into participants’ perceptions about the factors contributing to the poor outcomes in their respective hospitals. Areas explored with the participants included (1) perceptions regarding the factors that contribute to critical illness on admission and the link to earlier death on admission, (2) the reasons for premature discharge of patients and how this may influence nutritional recovery, (3) perceptions regarding the effectiveness of the WHO guidelines in improving treatment outcomes among patients admitted with SAM, including differential effectiveness by HIV status and disease stage, (4) possible challenges related to the implementation of the WHO guidelines and (5) other organisational and system-wide factors that may influence care and treatment outcomes for SAM in the study setting.

To optimise the credibility and trustworthiness of the study findings, participants who took part in both categories of focus groups were asked the same questions by the same facilitator. Member checking was conducted by means of reflection with participants to ensure that information they provided had been accurately understood. All the data were also collected and transcribed by one interviewer to improve trustworthiness.^25^

### Data analysis

All four interviews were transcribed verbatim in a Microsoft word processor. The interviews were transcribed separately so that the findings could be compared between the two hospitals. Data analysis followed Pope, Ziebland and Mays’s ‘framework analysis technique’^26^ which is geared towards generating policy and practice-oriented findings. The technique also has many advantages, including the fact that it preserves the integrity of individual responses throughout the analytical process, thereby providing a platform for reconsidering and reworking of ideas where more clarity is needed.^27^ The internal consistency of data coding and analysis processes were maximised by ensuring that one researcher did all the coding and analysis. However, a peer-review process was undertaken to validate the steps taken to analyse and interpret the data. This was important to ameliorate the ‘inter-coder reliability’ of the study findings.

### Ethical considerations

This study was approved by the University of the Western Cape Research Ethics Committee (Reg. no. 12/10/37).

## Results

The key themes that emerged during data analysis and the corresponding exploration domains are presented in Table 2.

**TABLE 2:** A breakdown of exploration domains and themes.

Exploration domain	Themes
1. Perceived reasons for critical illness on admission and the link with high hospital CFRs	a) The role of traditional medicine and household child negligence b) Poor disclosure rate of HIV status, poor adherence to antiretroviral therapy and their link to virological failure c) Misdiagnosis at the first point of care d) Lack of human resource capacity to deal with complex presentations and the high risk of death
2. Reasons for premature discharge and linkage to suboptimal nutritional recovery	a) Lack of hospital capacity to accommodate SAM cases during certain seasons b) Misguided perceptions about which discharge criteria to follow c) Lack of basic resources for preparation of feeds
3. Familiarity with the WHO guidelines and their perceived effectiveness	a) Periodic shortage of the health care workforce familiar with the WHO guidelines b) Perceived ineffectiveness of the guidelines in critically ill SAM cases c) Perceived equal effectiveness of the guidelines in HIV-positive and HIV-negative cases
4. Challenges related to the implementation of the guidelines	a) Changes in the skilled personnel to sustain quality of clinical care b) Sporadic shortage of therapeutic resources c) Organisational and system-wide challenges d) Inadequate staffing levels after normal working hours and higher CFRs

SAM, severe acute malnutrition; CFRs, case fatality rates.

### Perceived reasons for critical illness on admission and the link with high hospital case fatality rates

Being critically ill upon admission was perceived by many as a major risk factor for early death on admission. Many believed that there was a concordance of processes at household and community levels, which often culminate in a child being critically ill and admitted late to the hospital. There was a shared view that some parents and guardians of the children with SAM often consult traditional healers before bringing their child to the clinic or hospital.

‘My office is based in the paediatric ward, … parents tend to first seek traditional care, you know, and they (traditional medicine) become too severe for these children to handle [*interruption: Yes! – all agreeing*].’ (Participant 1, Social worker, Male, FGD 1)

‘…. What also happens is that although they may know that the child is HIV positive, they still have those beliefs that may be the child was bewitched or something, so they might not take treatment but may be take other routes [*traditional medicine*], I think that also might contribute to high deaths.’ (Participant 1, Clinical support manager, Male, FGD 2)

There was also a belief that the first visit to traditional healers often exacerbates the condition of the child to such a degree that by the time the child gets to the hospital, they are critically ill because of herbal intoxication and other fatal side effects associated with the use of traditional medicines.

‘So you can see the child has the signs of scarification or the signs of Sangoma visit and you ask when did you see the sangoma, they will say, when the child got sick.’ (Participant 1, Medical Officer, Male, FGD 1)

Prolonged gross negligence of the child was also another factor cited by participants and considered to be contributing to critical illness and eventual death upon arrival at the hospital. Participants believed that some mothers choose to leave the child under the custody of their grandmother who may not be fit and able to look after the child. Thus, the child’s condition may worsen over time in the absence of the mother.

‘… Sometimes even the parents are not there. The children are staying with the grandmother, … and the child will get worse because the grandmother does not have the resource and power to look after the child … so such things play a very big role in what we deal with here at the hospital.’ (Participant 1, Social worker, Male, FGD 1)

Participants argued that fatal paediatric cases have either been neglected by their mothers because of issues of stigma, or the mother does not feel comfortable enough to disclose the HIV status of the child to those who take care of the child in her absence. Also, sometimes when the HIV status of the child is diagnosed at hospital level, some mothers choose not to adhere to antiretroviral therapy (ART) for themselves and the baby.

‘I find that their mothers are very irresponsible. You will find that the child was born RVD [*retroviral disease*] positive. They will tell you that PCR was done at 6 weeks and when you ask them did you do a follow up to get the results, they will say that no and maybe they never even followed up on nevirapine, so the mothers can be very irresponsible. … It is especially the younger ones (interruption by nurses: yes, that is a problem).’ (Participant 2, Nurse, Female, FGD 2)

‘Another recurring theme in our hospital is lack of disclosure at home. … So, the mother knows she is RVD [*retroviral disease*] positive and the child has been exposed and she works away from home and she doesn’t disclose the status of the child to the family, so we have a lot of virological failures because she will only give the child ARVs because she herself is with the child, which will be intermittent.’ (Participant 3, Medical Officer, Male, FGD 2)

One clinician argued that there were some gaps in the quality of care provided at the first point of care, which are usually the primary health care clinics or the community health centres where children with SAM are first seen before they come to the referral hospitals. In the absence of the correct diagnosis for the child’s condition, the child gets worse over time, and by the time they are admitted to the hospital, they are critically ill and beyond resuscitation.

‘…When they come in, … they have been missed from the clinics. The diagnosis is found here and by that time the child is in bad condition as they will have been treated for the wrong condition.’ (Participant 3, Medical Officer, Male, FGD 2)

Lack of hospital capacity to deal with complex presentations was also linked to high CFRs for SAM. It was reported that some children with SAM present with complex conditions that a generalist medical officer may not easily detect or diagnose and that there was a lack of specialist physicians in the hospitals.

‘…But from a clinical perspective, one of the limitations is that because we are a level one hospital, and largely doctors who work in the paediatric ward are not paediatrician, we fail to pick up more complex presentations. So, if you find TB difficult to diagnose and disseminated fungal infection or certain signs of meningitis will also be missed, and so, the risk is that these RVD positive children are not being treated appropriately for complications. That may contribute to that earlier fatality.’ (Participant 3, Medical Officer, Male, FGD 2)

### Reasons for premature discharge and linkage to suboptimal nutritional recovery

The quantitative study revealed that, on average, the rate of weight gain achieved in both hospitals for cases admitted with SAM was suboptimal: < 5 g/kg/day–7.5 g/kg/day, which was far below the target of > 10 g/kg/day.^28^ Furthermore, SAM cases were often discharged without due regard for the discharge criteria. When asked the reasons for this practice, most HCWs argued that the decision to discharge SAM cases did not depend entirely on whether they had achieved sufficient weight gain. Some other factors were involved in the decision-making process, such as shortages of beds or even slight improvements in weight gain. This often led to premature discharge of the patient.

‘So what limits us is that during the dry season we have a lot SAM cases, and as soon as you have a number of the critical ones that’s when you move around the ward and say you look better you should go home and that is regardless of whether they have gained weight and are stable. But we do follow them up; the dietician will see them every two or six weeks after discharge.’ (Participant 4, Nurse, Female, FGD 2)

Another interesting finding was that the discharge criteria for SAM cases as indicated in the WHO guidelines were not consistently followed. Instead, the participants reported that they followed ‘national guidelines’ for discharging a patient. As shown in the quote below, there is a requirement by the hospital to discharge patients within 5 days of admission.

‘The thing is that we have national guidelines by which we have to comply because we don’t have to keep the patients, it should not be more than five days. It is a national norm.’ (Participant 4, Nursing service manager, Female, FGD 1)

Another reason mentioned for premature discharge was that sometimes there is no food to give the children and therefore it was considered pointless to keep them hospitalised.

‘Yes it would be a good idea to allow children to stay longer in the hospital if the resources are there, but because of lack of food, no milk, nothing – the mothers want to go home. … Even us we are not happy because there is no food to give them. It doesn’t make any difference if there is no food here and there is no food at home. So, it is better for them to go.’ (Participant 4, Nursing service manager, Female, FGD 1)

### Familiarity with the World Health Organization guidelines for the management of severe acute malnutrition and their perceived effectiveness

Some participants felt that the WHO guidelines for the management of SAM were useful and easy to implement.

‘I think in terms of the guidelines, especially the nurses, this is something we do quite well. They are straight forward [*yes, they are easy to follow! All in agreement*].’ (Participant 3, Medical Officer, Male, FGD 2)

This opinion was, however, contradictory to what emerged in a separate FGD with junior nurses at the same hospital. It was clear that even though the nurses had been in the ward for more than 6 months, they were not entirely comfortable with the use of the guidelines. In a follow-up question regarding the perceived usefulness of the guidelines, the nurses were asked whether they had been trained on how to use the guidelines and whether they were comfortable to use them. Three out of five nurses who were in the focus group were neither trained nor comfortable to use the guidelines.

Another dimension that emerged from participants around the effectiveness of the guidelines was that it was only helpful for cases that were not critically ill.

‘Sometimes we do get those cases like you mentioned those that die within 24 hours, they come in critical and then they end up dying with your initial treatment. So with the guidelines, the guidelines are implemented very well so I think it is one of those cases that will die anyway.’ (Participant 5, Dietician, Female, FGD 1)

It was interesting to learn that there were no differences in opinion regarding the relative effectiveness of the guidelines for HIV-positive children and their HIV-negative counterparts. Most FGD participants, particularly the nurses, seemed to be of the view that the guidelines should work well regardless of HIV status.

‘I don’t think the outcome would be different because of HIV infection: I think the outcome would be the same, if really we were at par with the training on SAM [*Interjection – Yeah! Most nurses agree*].’ (Participant 5, Ward nurse, Female, FGD 1)

### Some challenges related to the implementation of the guidelines

The perceived challenges related to the implementation of the WHO guidelines fell into three subthemes as follows:

- The changes in the skilled personnel to sustain quality of clinical care
- The sporadic shortage of therapeutic resources
- The organisational and system-wide challenges

One HCW stressed the importance of regular training opportunities being afforded to new or incoming staff, and posited that some months SAM cases are managed by relatively inexperienced clinicians (doctors and nurses) and who are not familiar with the WHO treatment guidelines, leading to poorer outcomes in those rotation cycles.

‘No the guidelines are working very well … it is just that may be in some intervals or period, training needs to be done, because there is rotation of nurses, sometimes there is also changes of doctors responsible for the ward. So, there needs to be constant training of nurses … I feel there is a gap there.’ (Participant 5, Dietician, Female, FGD 2)

Another challenge that emerged from the FGD was the sporadic shortage of resources such as required ingredients to prepare the starter and catch-up formula for the SAM cases to ensure timely and accurate feeding of these children. They reported that these shortages persisted in spurts over the years in both hospitals.

‘Sometimes we do not have mineral mix and some types of drugs or even ingredients for formula feeds. … So, they (Nurses) have to use their own money to buy for example milk or sugar.’ (Participant 6, Pharmacist, Female, FGD 2)

The participants were of the opinion that there was an inefficient and fragmented procurement system in both hospitals, with long delays in securing basic resources as a result of unnecessary bureaucratic channels through which most requests for supplies had to go.

‘We have ahhh, I don’t know … system problems. Delays when trying to approve the orders, service providers not complying or not being able to deliver what they are supposed to deliver even if the orders have been approved. … [*Interjection by one nurse, Yeah BEE – which stands for Black Economic Empowerment*]. Supply chain, that is where everything sort of gets stuck.’ (Participant 7, Nurse, FGD 1)

Inefficient communication channels between the hospital and the laboratory facilities were also reported as one of the organisational challenges. The laboratories serve as a support structure to the facility by assisting with the evaluation of blood samples and conducting other clinical examinations on patients’ specimens. According to HCWs, orders for blood tests are sent to the laboratory, but the results do not come back soon enough for the clinician to make crucial medical decisions.

‘The problem with the lab is that even though they operate in-house they tend to say that they have a problem with staff and they cannot process the results as efficiently as you would like, … by the time you get the results you are ready to send in another specimen for lab evaluation. So most of the time you are just treating them blindly hoping that they will recover.’ (Participants 1, Medical Officer, Male, FGD 1)

The quantitative phase of study revealed that more than 60% of the fatalities occurred after normal working hours.^10^ When participants were asked about what might have accounted for this pattern, some believed it was because there is poor staffing at that time, while others argued that it might be because front-line HCWs are too tired to attend to the patients who are in a critical condition. The two quotes below illustrate these divergent interpretations:

‘Maybe the kids are not getting feeds at this time because the nurses are relaxing, I don’t want to say they are sleeping but kids are not getting their treatment as they should.’ (Participant 4, Nursing service manager, Female, FGD 1)

‘You know the condition changes at night, so you know during the day it is better because you can get the doctor to come … I wouldn’t say that it is because the doctor is not called, it is just that may be there is no that urgency to call the doctor to come and see that child whose condition is changing.’ (Participant 5, Dietician, Female, FGD 2)

**FIGURE 1:**
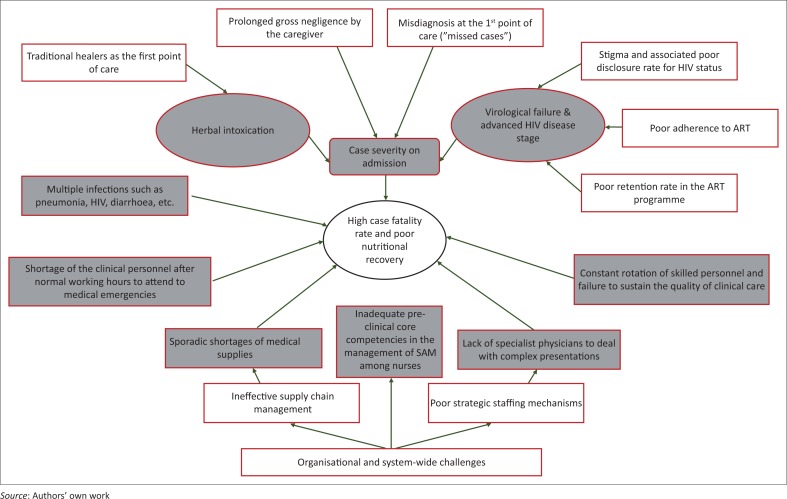
A framework of factors believed to contribute to high case fatality rates and poor nutritional recovery among children admitted with severe acute malnutrition in the study setting. Shaded boxes and circles represent proximal factors contributing to high case fatality rates and poor nutritional recovery, whereas the plain ones represent distal factors.

Figure 1 summarises the perceptions of HCWs around upstream and proximal factors that are believed to contribute to poor treatment outcomes among children admitted for SAM in the study setting.

## Discussion

This study described HCWs’ perceptions about factors that contribute, directly or indirectly, to high CFRs and poor nutritional recovery in children admitted and treated for SAM in two rural district hospitals in South Africa. The treatment of these cases followed the WHO 10-step guideline for the management of SAM. The aim of this study was to identify areas in the continuum of care which may require specific interventions to improve treatment outcomes for SAM in the study setting. A systematic pathway showing how these factors are believed by HCWs to act singly or synergistically to affect health care outcomes for SAM is shown in Figure 1.

Some of the findings from this study corroborate what has been reported in previous studies conducted in similar settings in South Africa. In this study, HCWs reported that the sporadic shortage of medical supplies – which was a direct result of ineffective supply chain management within the facility – contributed, in part, to poor health outcomes, particularly poor nutritional recovery. The HCWs also reported poor staffing strategies, lack of regular training and the lack of specialised physicians to deal with complex SAM presentation, as barriers to optimal health care delivery. A study conducted by Puoane et al.^29^ in a similar setting found that some of the factors that interfered with effective delivery of care for children admitted with SAM included lack of medical resources (e.g. electrolyte/mineral mix), inadequate professional training and a poor health system infrastructure. In a related study,^20^ it was established that the implementation of the guidelines can be feasible if nurses and doctors in rural hospitals are adequately trained to have the requisite expertise to effectively manage SAM cases. Karaolis et al.^30^ also demonstrated that the sustainability of the guidelines was affected by insufficient staff knowledge and inattentiveness to treatment protocols, and argued that regular training is a key determinant for effective health care delivery.

Ashworth et al.^18^ showed that regular training of paediatric staff, monitoring of the implementation of the WHO treatment guidelines for the management of SAM, and provision of support to the clinical team, and advocacy efforts with the hospital management teams can result in improved hospital level policies, quality of care and management systems, and consequently lead to improved treatment outcomes. A recent implementation science study conducted by Muzigaba et al.^22^ in the same study setting also showed that these improvements can indeed be realised through such interventions. However, the authors caution that the sustainability of such gains would require direct and continued involvement of an external party responsible for reinforcing the implementation of the intervention. Invariably, sustaining an intervention such as this necessitates a strong political will to mobilise the required human and financial resources as and when needed, as well as stakeholder commitment with a shared vision to improve health care outcomes.

The introduction of district clinical specialist teams (DCSTs) in the South African health care system holds promise in ensuring that there are sustainable mechanisms in place to ameliorate the continuum of care. There is a need to scale up this initiative in rural areas of the Eastern Cape Province where the deployment of DCSTs remains relatively sparse. Their mandate in conducting operations research and developing solutions as appropriate would play a huge role in improving leadership and accountability within the health care infrastructure.

Given the challenges reported in this and many other studies, in respect of shortages of HCWs who are adequately trained in the management of SAM, the need for curriculum infusion in the formal nursing and medical education system in South Africa is crucial. This is important considering the findings from this study that there is a concurrent policy in the study setting which mandates front-line nurses to rotate in different wards as part of the skills development initiative. Thus, all the nurses who undergo the clinical rotation within the paediatric ward should have the pre-clinical co-competencies on how to effectively manage cases with SAM using standardised WHO treatment guidelines.

This study also revealed additional community-based determinants of poor clinical outcomes that have not been extensively documented in previous studies. The HCWs reported having to occasionally manage SAM cases who have grossly been neglected by their caregivers. The HCWs argued that some SAM children are brought to the hospital with fatal comorbid complications arising from herbal intoxication. The treatment modality for such complications is not included in the WHO treatment guidelines used by HCWs in these facilities. This, coupled with the lack of specialised medical personnel, complicates the management of such cases with increased odds of death on admission. Furthermore, there are instances where children who have been infected with HIV default on their ART, mostly because of caregiver negligence, leading to virological failure and case severity, and eventually early death on admission. This view from HCWs is supported by empirical evidence from a related epidemiological study^10^ which showed that advanced HIV infection (Stage IV) and case severity were singly and interactively linked to a high probability of death on admission in SAM cases admitted in the same study setting. Case severity on admission was also reported by Maitland et al.^31^ as one of the strongest predictors of early death (death within 24 h of admission).

Given the above findings, it goes without saying that early detection of SAM would be a crucial step in preventing disease severity and the development of multiple SAM comorbidities. This would in turn reduce the likelihood of eventual preventable death associated with SAM. For children who develop SAM at home (community level), well-supported community-based nutrition rehabilitation programmes may play a huge role in alleviating the challenges associated with facility-based care such as the treatment of missed and severely ill SAM cases, high patient load and lack of resources, among other challenges. However, effective referral networks that link community-based therapeutic centres and the formal health care facilities would be needed, to ensure that cases that develop complications and require special attention are referred for specialised treatment. Community-based health workers can be identified and trained to take up this role. They may also be involved in efforts to integrate traditional medicine with the conventional health care system as recommended by the WHO.^32^ This approach could reduce the number of SAM cases with concurrent herbal intoxication who are admitted late at the hospitals in the study setting. Community-based health workers would also have a role to play in alleviating delayed care resulting from gross negligence by the caregiver of the child as was the case in this study.

This study has some limitations to note. Firstly, the transferability of the study findings is limited to the study setting, and possibly to the time when this study was carried out. Furthermore, the information gathered from the HCWs does not constitute the absence of additional evidences around factors that contribute to poor outcomes among children admitted for SAM in the study setting. The perceptions of caregivers of the patients seen at these facilities were not explored. Caregivers’ views would have added substantial value to the findings presented here. Furthermore, it is possible that HCWs who were interviewed may have given a biased account of factors that lead to poor outcomes, in favour of their practices and individual competencies. Thus, the findings presented here may need to be validated in other studies conducted in similar settings elsewhere.

## Conclusion

Findings from this study suggest that effective management of paediatric SAM in the study setting is affected by a multiplicity of factors that manifest at different levels of the health system, including organisational and system-wide policy and governance challenges; poor strategic staffing mechanisms; supply chain management issues; inadequate skilled medical personnel; poorly managed SAM comorbidities at baseline; base severity on admission, fragmented ART; misdiagnosis of SAM and related comorbidities at the first point of care; traditional medicine as the first point of care; and prolonged gross negligence of SAM cases by their caregivers. However, a verificatory study is encouraged to corroborate these findings.
